# Measurement of tissue azithromycin levels in self-collected vaginal swabs post treatment using liquid chromatography and tandem mass spectrometry (LC-MS/MS)

**DOI:** 10.1371/journal.pone.0177615

**Published:** 2017-05-12

**Authors:** Lenka A. Vodstrcil, Thusitha W. T. Rupasinghe, Fabian Y. S. Kong, Dedreia Tull, Karen Worthington, Marcus Y. Chen, Wilhelmina M. Huston, Peter Timms, Malcolm J. McConville, Christopher K. Fairley, Catriona S. Bradshaw, Sepehr N. Tabrizi, Jane S. Hocking

**Affiliations:** 1 Centre for Epidemiology and Biostatistics, Melbourne School of Population and Global Health, University of Melbourne, Parkville, Australia; 2 Murdoch Children’s Research Institute, Parkville, Australia; 3 Central Clinical School, Monash University, Melbourne Sexual Health Centre, Carlton, Australia; 4 Metabolomics Australia, Bio21 Institute, University of Melbourne, Parkville, Australia; 5 School of Life Science, University of Technology Sydney, Broadway, Australia; 6 University of the Sunshine Coast, Maroochydore, Australia; 7 Department of Microbiology and Infectious Diseases, The Royal Women’s Hospital, Parkville, Victoria, Australia; 8 Department of Obstetrics and Gynecology, University of Melbourne, The Royal Women’s Hospital Parkville, Victoria, Australia; Azienda Ospedaliera Universitaria di Perugia, ITALY

## Abstract

**Background:**

Azithromycin is recommended for the treatment of uncomplicated urogenital chlamydia infection although the standard 1gram dose sometimes fails to eradicate the infection (treatment failure). One hypothesis proposed for treatment failure has been insufficient levels of the antibiotic at the site of infection. We developed an assay using liquid chromatography and tandem mass spectrometry (LC-MS/MS) to measure azithromycin concentration in high-vaginal swabs and monitor how concentration changes over time following routine azithromycin treatment.

**Methods:**

Azithromycin concentrations were measured in two groups of women either within the first 24h of taking a 1g dose (N = 11) or over 9 days (N = 10). Azithromycin concentrations were normalised to an internal standard (leucine enkephalin), and the bulk lipid species phosphatidylcholine [PC(34:1)], using an Agilent 6490 triple quadrupole instrument in positive ionisation mode. The abundances of azithromycin, PC(34:1), and leu-enkephalin were determined by multiple reaction monitoring and absolute levels of azithromycin estimated using standard curves prepared on vaginal specimens.

**Results:**

Vaginal azithromycin concentrations of women were rapidly obtained after 5h post-treatment (mean concentration = 1031mcg/mg of lipid, range = 173-2693mcg/mg). In women followed for 9 days, peak concentrations were highest after day 2 (mean concentration = 2206mcg/mg, range = 721-5791mcg/mg), and remained high for at least 9 days with a mean concentration of 384mcg/mg (range = 139-1024mcg/mg) on day 9.

**Conclusion:**

Our study confirmed that a single 1g dose of azithromycin is rapidly absorbed and remains in the vagina at relatively high levels for at least a week, suggesting that poor antibiotic absorption is unlikely to be an explanation for treatment failure.

## Introduction

*Chlamydia trachomatis* is the most commonly reported bacterial sexually transmitted infection (STI) in developed countries, with over 1.4 million cases reported in the United States in 2012 [1]. Left untreated, chlamydia can ascend from the endocervix to the upper genital tract in women and cause pelvic inflammatory disease (PID), which can increase the risk of developing fallopian tube scarring, potentially leading to ectopic pregnancy, tubal infertility and chronic pelvic pain [2–4]. Current first-line treatment for uncomplicated urogenital chlamydia in the United states (US) [5], Europe [6] and Australia [7] is a single 1g dose of azithromycin or seven days of doxycycline (100mg twice daily).

Azithromycin is rapidly absorbed from the gastrointestinal tract following oral administration, with peak serum concentrations measured within 2-4h [8]. Azithromycin is then widely distributed and concentrated into body tissue including within cells, resulting in high tissue concentrations where it is slowly released, resulting in a long terminal phase elimination half-life of 68h [9], making it suitable to be used as a single dose treatment.

Recently however, the effectiveness of a single-dose of 1g azithromycin for the treatment of genital chlamydia has come under debate [10–14], with increasing reports of repeat chlamydia infections following treatment. While most repeat infections are likely to be re-infections, emerging evidence suggests that treatment failure with azithromycin may account for a proportion of these. Studies have reported treatment failures occurs in about 8% of women following 1g azithromycin in whom the risk of re-infection has been ruled out [15, 16], and a recent meta-analysis found that the efficacy of azithromycin for the treatment of urethral or cervical chlamydia infection may be 94%, lower than the 97% previously reported [17].

Although azithromycin levels are thought to remain well above the reported minimum inhibitory concentration (MIC) for chlamydia in genital tissues and mucus for 10 to 14 days after a 1g dose [18, 19], there are only limited data available and none in recent years when concern about azithromycin treatment failure has been increasing.

The objective of the current study was to develop an assay to quantify the concentration of azithromycin in self-collected high-vaginal swabs and blood specimens using liquid chromatography and tandem mass spectrometry (LC-MS/MS) [20–22] following treatment. In the light of recent concerns about the efficacy and absorption of azithromycin, we wanted to determine if azithromycin is being delivered to the site of infection and establish how concentrations change over time. Our primary outcome was to establish azithromycin concentrations in vaginal tissue over an A) 24h and B) nine day period and our secondary outcome was to determine plasma azithromycin concentrations 4h post-dose. This paper describes the assay and our results.

## Methods

### Recruitment of participants

**Fig 1:**
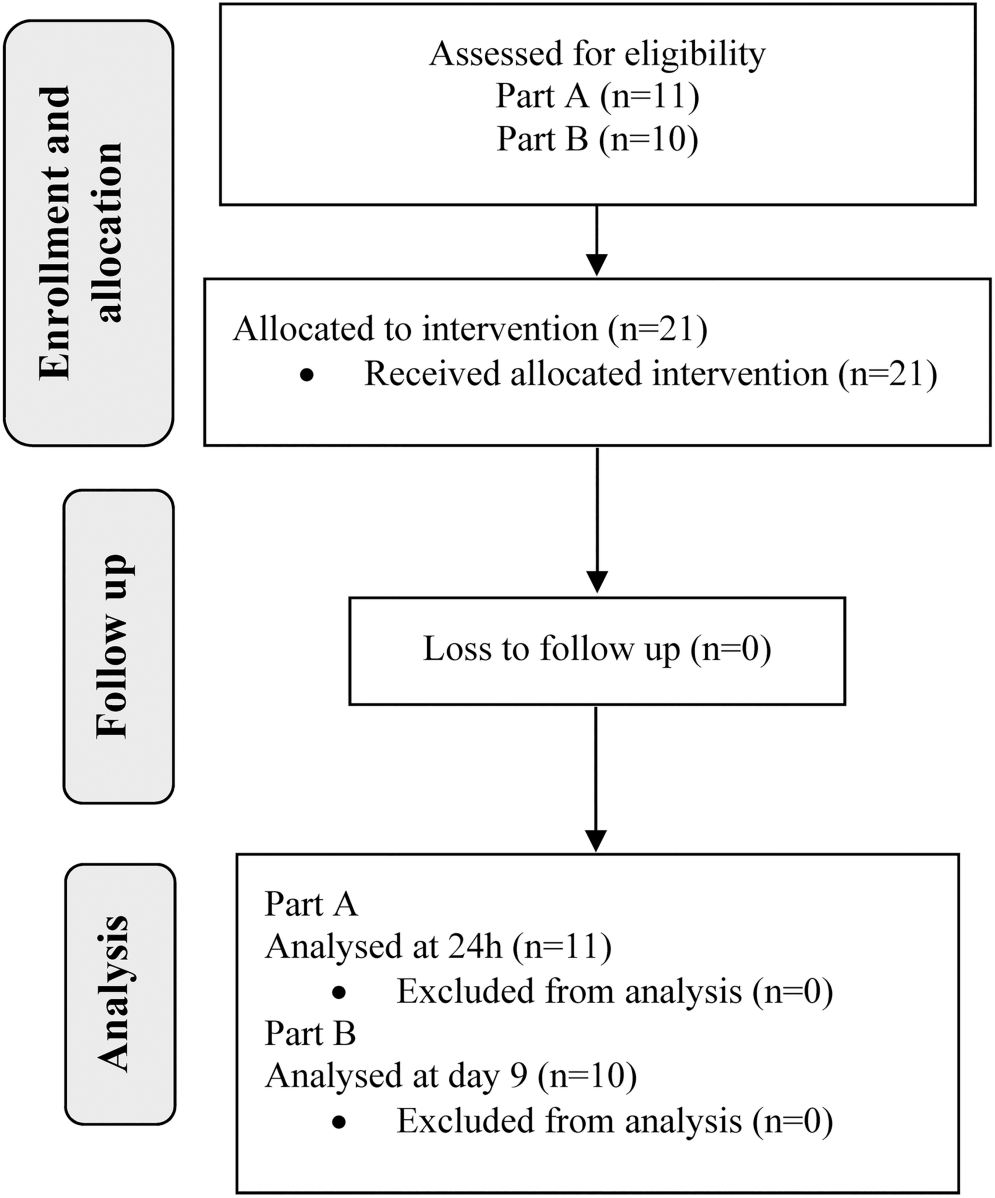
Flow chart indicating different phases of the study.

The study comprised two parts using self-collected vaginal swabs (FLOQSwab™, Copan diagnostics, Interpath Services PTY LTD, Heidelberg West, VIC, Australia): Part A incorporated repeated sampling over the first 24h post azithromycin treatment, and Part B involved repeated sampling over nine days following treatment. In Part A and B, a convenience sample of 11 and 10 women without chlamydia, respectively, were recruited from the University of Melbourne and the Melbourne Sexual Health Centre (MSHC) between January 2013 and June 2014 through advertising on a University of Melbourne staff email list and word of mouth (Fig 1). These recruitment sites allowed for collection and rapid processing of specimens by the research team located at MSHC.

#### Participant eligibility, participant data and specimen collection

Women were eligible for inclusion if they were aged 18 years and older, could speak English, had no known allergies to azithromycin or any of its components, were not taking any contra-indicated treatments nor had taken any azithromycin in the last 6 weeks, were not pregnant, were not currently menstruating, and were able to attend the clinic and provide all the required swabs over the study period. A research nurse explained the study to each participant, obtained informed written consent and instructed them how to self-collect a high vaginal swab (swab contains nylon fibres; FLOQSwab™, Copan diagnostics). Women were asked if they were using any intra-vaginal gels or creams prior to the study and to record any adverse effects including diarrhoea and stomach cramping. After collecting their initial swab, all participants took the intervention, a prescribed treatment of a single 1g dose of azithromycin, in the presence of the research nurse for the purposes of this study and to facilitate assay development. In Part A, 11 women each self-collected a swab every 30 mins for the first five hours after taking the treatment and then provided a final swab 24h after taking the treatment (total of 12 swabs, including swab prior to taking the treatment [baseline swab]). In Part B, a different group of ten women collected a swab every 24h for nine days after taking the treatment (total of 10 swabs including swab prior to taking the treatment [baseline swab]). In both Parts, women provided a blood specimen at 4 hours after taking the treatment. There were no protocol deviations, no participants were lost-to-follow-up and no comparator groups. Participants received a reimbursement for their time in the form of a shopping voucher upon completion of all study requirements ($75 for Part A or $100 for Part B).

High-vaginal swabs were immediately agitated in 1ml of cold 100% methanol (MeOH) following collection and kept at -80°C for up to a month until analysis. All high-vaginal swabs and plasma specimens from both components were analysed using liquid-chromatography tandem mass spectrometry (LC-MS/MS).

#### Study registration

The study was prospectively registered using a Clinical Trial Notification form lodged with the Australian drug regulatory authority, the Therapeutic Goods Administration, in compliance with the requirements of the Alfred Hospital Human Ethics Committee. It was retrospectively registered with the Australian New Zealand Clinical Trials Registry (ANZCTR; ACTRN12616001684415). The authors confirm that all ongoing and related trials for this drug/intervention will be registered.

#### Ethics approvals

Ethical approval for this study was granted by the Alfred Hospital Human Ethics Committee (Project No 480/11) and registered with The University of Melbourne Central Human Research Ethics Committee (Health Science Human Ethics Sub Committee; Project No 1238244).

### Liquid chromatography-mass spectrometry

#### Instrument system and conditions

Azithromycin and phospholipids were detected and quantified using a Agilent 1290 LC system (comprising a binary pump, degasser, auto samples and column heater) connected to an Agilent triple quad (QQQ 6490) mass spectrometer. Azithromycin was resolved on an Agilent Porshell 120 SB-C18 (2.7μm) 2.1 x 100mm column. Samples were eluted using water-acetonitrile-MeOH with 20mM ammonium acetate as a mobile phase at the flow rate of 0.5mL/min. The mobile phase gradient started at 5% of acetonitrile: MeOH (1:1/ (v/v)), was held for 1 min, then linearly increased up to 100% in 5 minutes, held for 4 min, then equilibrated for 3 mins as the initial condition. The detection of azithromycin and the internal standard, leucine enkephalin (Sigma Aldrich), was performed in tandem mass spectrometry operated in positive ion electrospray ionisation and multiple reactions monitoring (MRM) mode using the transition of *m/z* 749 → 592 and 556 → 397 for azithromycin and leu-enkephalin, respectively. Leu-enkephalin concentrations were measured to determine consistency in specimen preparation across samples and runs indicating consistency. The parameters of the mass spectrometer for azithromycin analysis were: capillary voltage of 4000V, fragmentor voltage of 380V and collision energy of 60V. The sheath gas temperature was 350°C and the collision gas was nitrogen (N_2_) at 7L/min.

Extracted total phospholipid (5μL) aliquots were resolved on a 50mm × 2.1mm × 2.7μm Ascentis Express RP-Amide column (Sigma-Aldrich Supelco) using an Agilent LC 1200. Samples were eluted at 0.2mL/min over a 5 min gradient of Milli-Q® water/ MeOH / tetrahydrofuran (50:20:30, v/v) to water/ MeOH/ tetrahydrofuran (5:20:75, v/v), with the final buffer held for 3 min. Separated lipids were analysed by electrospray ionisation-mass spectrometry using an Agilent QQQ 6460. An MRM transition of *m/z* 760 → 184 was used to quantify the lipid species of phosphatidylcholine [PC(34:1)], as a marker of bulk phospholipid and cellular material.[23] The capillary voltage, fragmentor voltage, and collision energy were 4000V, 140 – 380V, and 15–60V, respectively. The collision gas was N_2_ at 7L/min.

The extraction efficiency of azithromycin from vaginal swabs and plasma specimens was calculated to establish the extraction protocol and to ensure that the protocol provided efficient extraction of azithromycin from specimens analysed (see S1 File for extended methodology). The extraction efficiency of azithromycin for vaginal swabs and plasma was found to be 80.3% (+/- standard deviation [SD] 2.1%) and 89.5% (+/- SD 4.3%), respectively.

#### Absolute azithromycin recovery, precision and accuracy

The analytical recovery of azithromycin was determined at concentrations of 5, 50 and 500ng/mL by comparing the peak areas of azithromycin detected from drug-free matrix (either vaginal swabs or plasma specimens). Drug-free matrix was spiked with azithromycin standards prepared in 100% MeOH. The absolute recovery of azithromycin was 78% and 88% for vaginal and plasma specimens, respectively.

The azithromycin was detected in both plasma and vaginal material, with a lower detection limit of 10ng/mL and linear range of 10-1000ng/mL for the azithromycin standard prepared in both plasma and vaginal material.

Accuracy and precision of the method was evaluated by analysing the same sample of azithromycin prepared in the presence of the vaginal cell matrix multiple times within a batch. The within-batch precision was 2.1% of the relative standard deviation (RSD). The accuracy of the method between batches was determined by analysing the same sample over 24h and RSD was found as 9%.

#### Selectivity, stability & linearity

The selectivity of the analytical method was evaluated by analysing azithromycin-free plasma samples and baseline vaginal swabs. No azithromycin was detected in the drug-free plasma or the baseline vaginal swabs providing evidence of the selectivity of our analytical methods. Our study was unlikely to suffer from any issues from stability because azithromycin has been previously demonstrated to be stable at room temperature for 115h[22] and at 4°C for 25 days,[24] and azithromycin in human plasma stored at -20°C has been found to be stable over five freeze-thaw cycles.[22]

#### Participant specimen preparation

For analysis of participant specimens, all stored vaginal specimens in 100% MeOH were processed in 1mL of chloroform (CHCl_3_) containing 1ug/mL leu-enkephalin, and azithromycin was extracted from cellular material as described above. The organic layer was then dried under a gentle stream of N_2_ at 40°C. The residue was reconstituted in 100μl of 100% MeOH. Absolute quantitation of azithromycin present in the specimens was carried out using azithromycin standards (see below) prepared on vaginal material swabs, which has taken from participants before the drug administration. Lipid concentration was also determined to normalise for differences in swab or plasma collection both within and between participants, as described below.

#### Plasma sample preparation

Plasma samples (0.05mL aliquots) were transferred to Eppendorf tubes and azithromycin was extracted by adding 1mL of MeOH followed by 1mL of CHCl_3_ containing 1 microgram(mcg)/mL leu-enkephalin. Samples were vortex mixed for 1 min, agitated for a further 30 min at 30°C on a mechanical thermo-shaker and then centrifuged at 15,000 *× g* for 15 mins to separate phases. The lower organic layer was then dried under a gentle stream of N_2_ at 40°C and the residue reconstituted in 100% MeOH (100μL). Absolute quantitation of azithromycin present in the plasma was carried out using azithromycin standards prepared on blank plasma.

#### Preparation of azithromycin standards & azithromycin concentration calculation

Both an azithromycin tablet and pure azithromycin (Sigma Aldrich) were used to generate the standard curves in spike biospecimens using a dose series and these results were used to normalise the participant samples (see S1 File for extended methodology).

#### Pharmacokinetic analysis

For all samples and standards, LC-MS data were processed using the Agilent MassHunter quantitative software (Agilent Technology, version 5). Calibration curves were generated by assaying the above azithromycin standard curve samples. The linearity of each calibration curve was determined by plotting the nominal concentration of azithromycin to the peak area ratio of azithromycin, normalised to leu-enkephalin and lipid concentrations of PC(34:1) using the following formula:

$$Normalised\;peak\;area\;of\;azithromycin\;in\;an\;individual\;sample\;=\;\frac{\left[a(AZ)\;x\;A(IS)\;x\;A(L)\right]}{\left[a(IS)\;x\;a(L)\right]}$$

Where, a(AZ) = the peak area of azithromycin in the tested sample; A(IS) = the average peak area of leu-enkephalin (internal standard) in all specimens; A(L) = the average peak area of 5μM lipid species [PC(34:1)] in all specimens; a(IS) is the peak area of leu-enkephalin in the test sample; and a(L) is the peak area of lipid species PC(34:1) in the tested sample. The lower limit of detection was defined as three times the noise peak and determined to be 10ng/mL in matrix.

Azithromycin levels for each specimen provided by the participants were calculated using the peak area of azithromycin, PC(34:1) and leu-enkephalin, respectively. The peak area of azithromycin was then normalised to leu-enkephalin and lipid content using the above equation. The concentration of azithromycin was calculated using the normalised peak area of azithromycin and the standard curve prepared for azithromycin in the blank vaginal material specimens. Plasma concentrations of azithromycin were calculated using the peak area of azithromycin and are expressed as ng/mL of plasma.

### Statistical analysis

Descriptive statistics (mean and range) are reported unless indicated otherwise. Results are presented as mean normalised concentration of azithromycin as mcg/mg of lipid standard. For analysis of the normalised azithromycin concentrations, data were log transformed and linear regression was used to determine differences in azithromycin concentrations over time in both Part A and B separately, due to the two experimental runs. Statistical analyses were performed using Stata/IC (Version 14, StataCorp LP, College Station, USA).

## Results

Results are published in accordance with the Transparent Reporting of Evaluations with Non-randomised Designs (TREND) statement (see S2 File for checklist) [25].

### Patient demographics

In Part A, eleven women, aged 24–38 years (median = 30), provided a total of 12 vaginal swabs each over the course of 24h following a 1g dose of azithromycin. For Part B, 10 different women aged 20–48 (median = 34) provided a total of 10 vaginal swabs each over the course of 9 days following a 1g dose. None of the women currently used any intra-vaginal gels or lubricants.

### Azithromycin concentrations within 24h post-dose (Part A)

**Fig 2:**
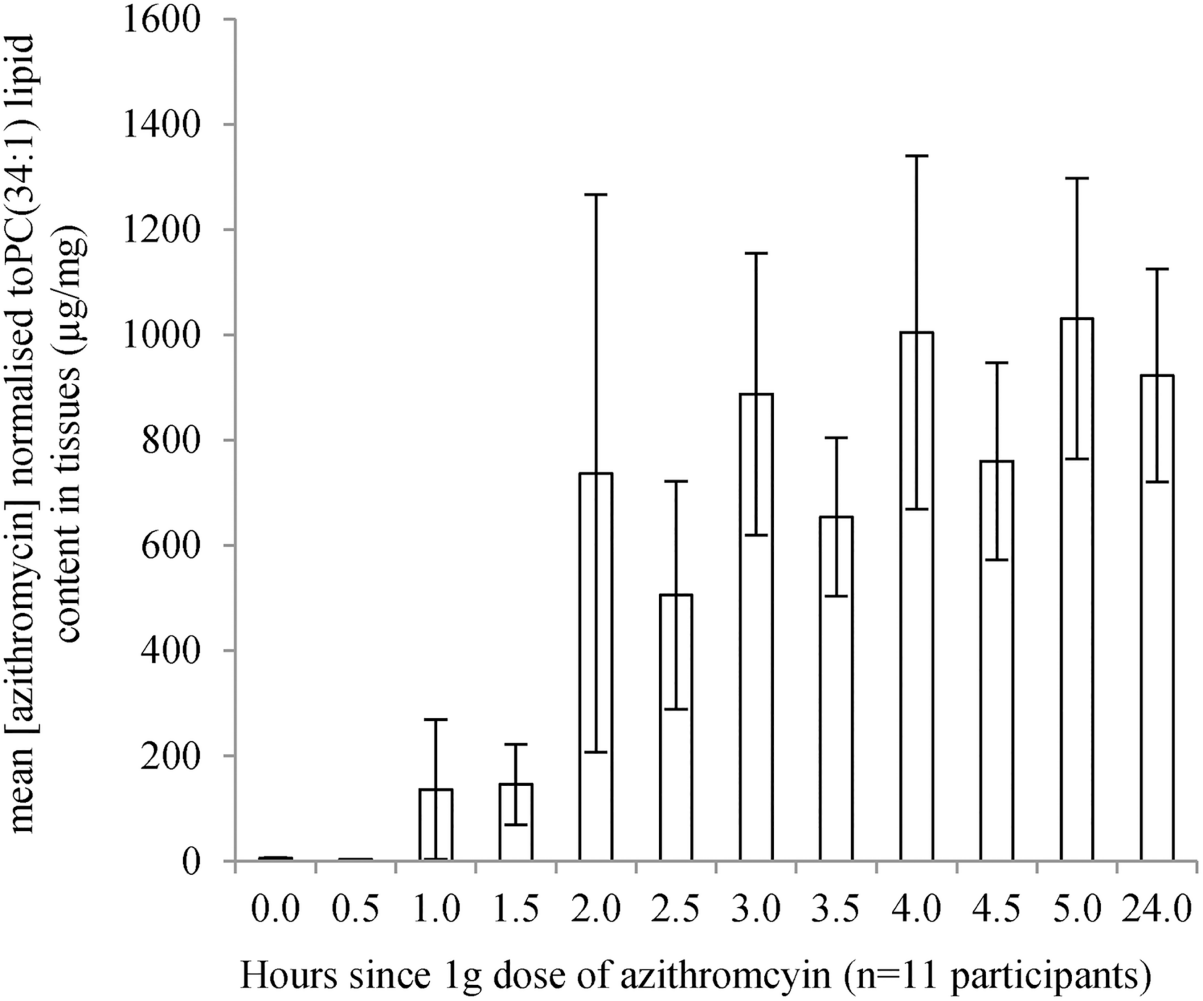
High vaginal azithromycin concentration over 24h. Mean (+/- standard error of the mean) normalised concentration of azithromycin in the vagina as mcg/mg of lipid content, PC(34:1) in tissues for each time point up to 24h after a single 1g dose of azithromycin in 11 participants.

Azithromycin was detected in all 11 women with the internal control concentrations, leu-enkephalin, remaining consistent across samples and runs indicating consistency in specimen preparation. Within one hour of taking azithromycin, the mean normalised concentration of azithromycin was 136mcg/mg of lipid (range = 1-1463mcg/mg) and significantly increased (beta coefficient = 0.57; 95%CI = 0.54, 0.64, p <0.001) to reach the highest mean concentration 5h post-dose of 1031mcg/mg (median = 733mcg/mg, range = 173-2693mcg/mg) (Fig 2). The mean plasma concentration of azithromycin at 4h was 915ng/mL of plasma (median = 908mcg/mg, range = 388-1406ng/mL) demonstrating that the antibiotic had been absorbed for each study participant. None of the 11 women reported any diarrhoea but three reported abdominal cramping and two were nauseous 2-4h after taking azithromycin. There was no pattern between reporting a side effect and azithromycin concentration over the period analysed.

### Azithromycin concentrations up to 9 days post-dose (Part B)

**Fig 3:**
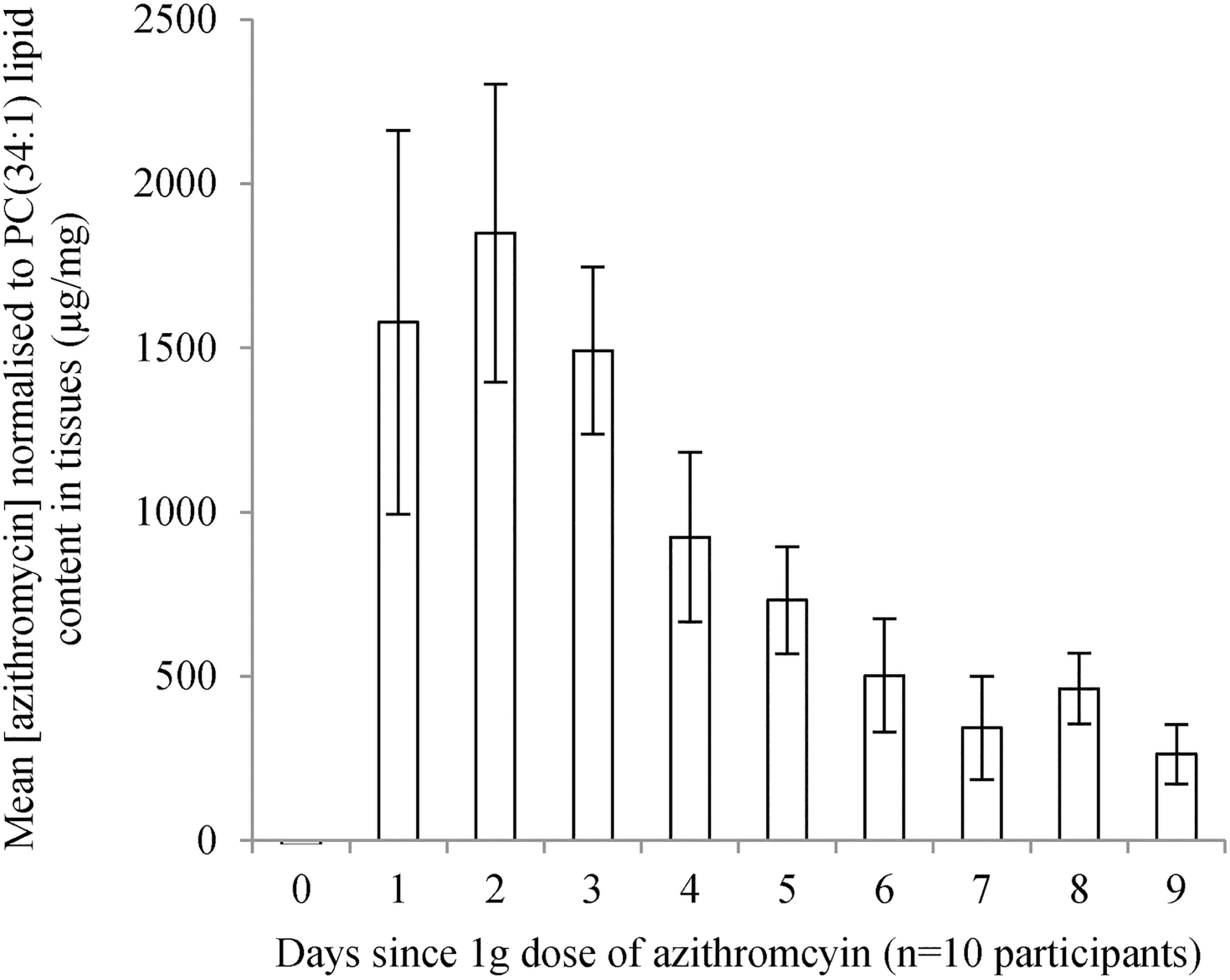
High vaginal azithromycin concentration over 9 days. Mean (+/- standard error of the mean) normalised concentration of azithromycin in the vaginal as mcg/mg of PC(34:1) lipid content in tissues for each time point up to 9 days following a single 1g dose in 10 participants.

Azithromycin was detected at varying concentrations in all 10 women post-treatment. Within two days the mean normalised azithromycin concentration in vaginal material was highest, with a concentration of 2206mcg/mg of lipid (median = 1850mcg/mg, range = 721-5791mcg/mg) then significantly decreased over time (beta coefficient = -0.16; 95%CI = -0.26, -0.05, p = 0.010) with the lowest mean azithromycin concentration of 384mcg/mg (median = 890mcg/mg, range = 139-1024mcg/mg) on day 9 post-treatment (Fig 3). The mean plasma concentration of azithromycin was 930ng/mL (median = 1031mcg/mg, range = 408-1518ng/mL) 4h post-dose. Six of the 10 women reported diarrhoea and an additional one woman reported abdominal cramping. The six women reporting diarrhoea were more likely to have lower peak concentrations 24 hours post-dose, but no associations with lower concentration over time were observed. The lowest concentration on day 9 was reported by a woman who experienced no side effects from the azithromycin.

## Discussion

We have developed an assay for detecting azithromycin in self-collected high-vaginal samples using LC-MS/MS. Following a 1g dose of azithromycin, high vaginal concentrations were rapidly obtained within 5 hours post dose, and in women followed for 9 days, peak concentrations were reached within 48 hours, remaining high for at least 9 days. This timeline is important because of the 48-72h developmental cycle of chlamydia [26] and means that the organisms in the vagina will be exposed to an effective dose during the organism’s developmental cycle.

Treatment efficacy in women has been questioned [10, 12] but our findings, though limited to a small number of women, suggest that treatment failure in vaginal infection is unlikely to be due to poor absorption of the antibiotics [27]. Chlamydia persistence whereby the chlamydia organisms are viable but non-infectious and may require greater doses of azithromycin to effectively kill [28, 29], is an alternative mechanism for treatment failure. It has been demonstrated *in vitro* that chlamydia, under the selective pressure of beta-lactam antibiotics [30], interferon-gamma (IFN-Ƴ) or deprivation of nutrients, such as iron or amino acids (e.g. tryptophan), can enter a persistent, metabolically inactive state [31, 32] and is less sensitive to azithromycin. This may be especially relevant in populations with a higher risk of exposure to other sexually transmitted infections requiring treatment with beta-lactam antibiotics.

In our study, diarrhoea was reported by about one quarter of the women within 2-4h of taking a 1g dose of azithromycin. Diarrhoea could result in low tissue concentrations due to poor drug absorption, however all women still reached high concentrations of azithromycin regardless of whether or not they reported diarrhoea. Nevertheless, poor drug absorption from the gut as a result of diarrhoea may directly correlate with tissue concentrations and should be considered when assessing treatment efficacy.

Another potential mechanism leading to treatment failure includes the possibility that chlamydia has developed macrolide resistant mutations. Although chlamydia isolates from suspected failures have been demonstrated to have multi-drug resistance *in vitro* [33–38] with mutations in the 23S rRNA gene described [39, 40], this has not been demonstrated *in vivo* and there has been no recent data demonstrating decreased antimicrobial sensitivity over time.

### Limitations

We used self-collected high vaginal swabs in this study as they are acceptable to participants and provide similar results as clinician collected samples [41]. However, sampling variability within and between women could affect the amount of cellular material available for analysis and impact on results. To minimise this limitation, we normalised the variability in specimen collection by estimating the number of cells collected using the concentration of lipid species PC(34:1) present within each specimen. The specimens provided were from women who did not have a chlamydia infection. As a result, we cannot make any comment about whether azithromycin is absorbed differently between women who do or do not have a chlamydia infection. However studies have shown that azithromycin concentrations are higher in tissues that are inflamed raising the possibility that concentrations may be higher in the presence of chlamydia infection [42–44]. Furthermore, our results may not be generalizable to the larger population due to the small sample size used for assay development. Unfortunately the same women were not included in both the 24h and 9 day components as a result of study logistics. However, any measurement bias between the two components was minimised by calculating the absolute quantification of azithromycin using an azithromycin standard curve on each run and again, by normalising the azithromycin concentration to the number of cells present.

A comprehensive evaluation of *Chlamydia trachomatis* MIC reported it as 0.125mcg/g [45] and the levels reported in our study exceeded this concentration. However, assessing antimicrobial efficacy by comparing tissue concentrations (from homogenates) with MIC is not reliable, as the pharmacokinetics of antibiotics is complex [46]. Pharmacokinetic studies have shown that high intracellular concentrations are very typical for azithromycin, as levels rapidly decrease in the blood within 24 hours post dose suggesting most of the drug is absorbed into tissue [47, 48]. Azithromycin has also been reported to be distributed in mucus as well as in tissue, which is likely to also contribute to its efficacy [19]. One early study measured azithromycin concentrations in cervical mucus (collected by sucking the cervical canal with a syringe tipped catheter) and found detected levels for 14 days following a single 1g dose [19].

Our measured drug concentrations will represent both protein-bound and protein-unbound (free) drug. Free drug concentrations may be more important as this is the pharmacologically active form [49, 50] where the drug is able to enter cells and exert its anti-chlamydial effect. Despite this, protein binding for azithromycin is low and concentration dependent, decreasing from 51% at 0.02mcg/mL to 7% at 2mcg/mL [8], which suggests in the high concentrations observed in our study, most of the drug would be expected to be “free” drug. Future studies might employ methods that allow isolation of free drug (e.g. microdialysis) to provide more precise measurements of free drug [51]. Despite these limitations, we have developed an assay that is able to detect and quantify the concentration of azithromycin in self collected vaginal specimens and demonstrate that azithromycin is being delivered to the site of infection.

### Conclusions

By using self-collected vaginal swabs, we were able to demonstrate that a single dose regimen of 1g of azithromycin is rapidly absorbed and remains at high concentrations in the vagina for at least a week and that poor antibiotic absorption is unlikely to be an explanation for treatment failure. We have developed a laboratory assay for measuring azithromycin concentrations in self-collected vaginal swabs. This assay could be used for estimating azithromycin concentration in high-vaginal material collected from women who have suspected treatment failure.

## Supporting information

S1 File Extended methodology. (DOCX)

S2 File TREND statement checklist. (DOCX)

S3 File Clinical trial protocol 1. Clinical trial protocol submitted to and approved by the Alfred Hospital Ethics Committee.
(PDF)

S4 File Ethics approval certificate 1. Alfred Hospital Ethics Approval Certificate for Clinical Trial Protocol 1.
(PDF)

S5 File Clinical trial protocol 2. Amended clinical trial protocol submitted to and approved by the Alfred Hospital Ethics Committee (labelled version 2).
(PDF)

S6 File Ethics approval certificate 2. Alfred Hospital Ethics Approval Certificate for amended Clinical Trial protocol 2 (version 2).
(PDF)

S7 File Clinical trial protocol 3. Second amended clinical trial protocol submitted to and approved by the Alfred Hospital Ethics Committee (labelled version 5).
(PDF)

S8 File Ethics approval certificate 3. Alfred Hospital Ethics Approval Certificate for amended protocol 3 (labelled version 5).
(PDF)
