# Supplementary material for: Measurement of tissue azithromycin levels in self-collected vaginal swabs post treatment using liquid chromatography and tandem mass spectrometry (LC-MS/MS)
Source: PLoS One. 2017 May 12;12(5):e0177615. doi: 10.1371/journal.pone.0177615 (PMC5428968; doi:10.1371/journal.pone.0177615)
Supplement: S1 File — (DOCX) [file pone.0177615.s001.docx]

Supplementary material 1: Extended Methodology

*Extraction efficiency*

We determined the extraction efficiency of azithromycin from vaginal swabs and plasma specimens separately. Firstly, we validated the use of Copan FLOQ swabs (Interpath Services, Heidelberg West, VIC, Australia) for their use to detect azithromycin. Three swabs were swirled in 1mL of 100% MeOH then spiked with 5μL of 100μg/mL azithromycin (*Recovery* swabs; Sigma Aldrich). At the same time, six swabs were swirled in 1mL of 100% MeOH; three for use as blank controls with no azithromycin added (*Blank* swabs), and three of which had azithromycin added post-exaction (*Control* swabs). All specimens were then processed in 1mL of chloroform (CHCl_3_; B&J chemicals) for specimen extraction containing 1ug/mL leucine enkephalin. The organic layer was dried under a gentle stream of nitrogen at 40^o^C. The residue in the *Recovery* and *Blank* swabs was reconstituted in 100µL of 100% MeOH. The residue in the *Control* swabs was reconstituted with 95μL of MeOH and then spiked with 5μL of 100μg/mL azithromycin. Specimens were processed on the Agilent QQQ as described in the manuscript. The extraction efficiency was calculated by comparing the peak area of azithromycin detected from *Recovery* swabs to *Control* swabs and determined to be 80.3% (+/- standard deviation [SD] 2.1%).

*Plasma specimen extraction efficiency*

Six aliquots of 50µL drug free human plasma were spiked with 5μL of 100μg/mL azithromycin and then quenched with 1000µL of 100% MeOH (*Recovery* plasma). At the same time, twelve aliquots of 50µL drug free human plasma were quenched with 1mL of 100% MeOH, without azithromycin, six for use as blank controls (*Blank* plasma), and six of which had azithromycin added post-exaction (*Control* plasma). All samples were processed as described in the manuscript. The extraction efficiency was calculated by comparing the peak area of azithromycin detected from *Recovery* plasma to *Control* plasma samples and determined to be 89.5% (+/- SD 4.3%).

*Preparation of azithromycin standards & azithromycin concentration calculation*

We used two azithromycin standards to develop standard curves: one using 10mg/mL of pure azithromycin that was commercially available (Sigma Aldrich; standard 1), and the second using a 500mg azithromycin tablet (standard 2) as taken by participants. Two standards were used so that a correction factor could be derived to validate the detection of azithromycin in tablet form against the pure azithromycin in Part B of the study (9-day study) when only the tablet-derived standard curve was used. To dissolve the tablet for standard 2, a 500mg azithromycin tablet weighing 942mg was powdered using a motor and pestle, and then 559mg of powder dissolved in 29.6mL of 100% MeOH to prepare an equivalent concentration of 10mg/mL stock azithromycin solution. This was then filtered through MILLEX-GP 0.22µm PES membrane (Merck Millipore).

A sample matrix (vaginal cellular material) was used to serially dilute both azithromycin standards. Two additional volunteers who did not receive azithromycin self-collected a total of 18 high-vaginal swabs to provide vaginal cellular material that did not contain any azithromycin. Nine of these samples from the two women were spiked with pure azithromycin (standard 1) and the second nine samples were spiked with a dissolved azithromycin tablet (standard 2), both at serial concentrations ranging from 0.1-1000 ng/mL.

The recovery of azithromycin from the tablet after the filtration step was corrected using the response difference between the two standard curves of azithromycin. The detection of azithromycin in tablet form was validated against the pure azithromycin to derive a correction factor of 1.2 that was used to correct the azithromycin concentrations calculated in Part B of the study.
