# Supplementary material for: Measurement of tissue azithromycin levels in self-collected vaginal swabs post treatment using liquid chromatography and tandem mass spectrometry (LC-MS/MS)
Source: PLoS One. 2017 May 12;12(5):e0177615. doi: 10.1371/journal.pone.0177615 (PMC5428968; doi:10.1371/journal.pone.0177615)
Supplement: S3 File — Clinical trial protocol submitted to and approved by the Alfred Hospital Ethics Committee. (PDF) [file pone.0177615.s003.pdf]

Protocol

Aim: To determine a standard specimen collection and testing method for measuring azithromycin concentrations in cervical cellular material in women following a single 1 gram dose.

Rationale:

- There is considerable concern internationally about chlamydia treatment failure.[1-7]
- Studies have found that chlamydia treatment failure may be at least 8%,[8 9] considerably higher than the 2-3%[10] failure expected based on results of previous chlamydia treatment trials that with the exception of one trial, all used the less sensitive culture to measure antimicrobial cure.
- A treatment failure rate of 8% rather than 2-3% means a further nearly 3,000[11] in Australia and 70,000[12] women in the USA were inadequately treated for chlamydia in 2009, leading to longer duration of infection, increased risk of developing sequelae and continued transmission.
- **It remains uncertain if this treatment failure is because of (1) re-infection by an untreated partner; (2) failure of the drug itself due to poor absorption from the stomach or low concentrations in female genital tissue where it is needed to work (3) the body's immune system's inability to fight the infection.**
- We have received NHMRC funding to conduct a large cohort study of 450 women that aims to estimate azithromycin treatment failure for genital chlamydia infection. In order to understand failure due to (2) above (poor absorption), we propose to develop a test to measure the concentration of the drug in female cervical cellular material. We wish to determine whether azithromycin is present at the cervix in sufficient concentrations to kill chlamydia. This was done by Worm and colleagues in the early 1990s1 on mucus aspirated from the cervix, a method unlikely to be acceptable to women today as part of a large cohort study.
- **The purpose of this study is to develop a test and specimen collection method that measures azithromycin absorption in cervical cellular material (mucus and cells) using a specimen that is practical and acceptable to women – vaginal swabs or tampons.**

Background:

Chlamydia rates continue to increase

Worldwide, chlamydia diagnosis rates have increased dramatically over the last decade – it is the most commonly diagnosed bacterial STI throughout the developed world. Chlamydia diagnosis rates have increased by over 400% in the last decade in Australia with 72,123 cases diagnosed in 2010.[11] Part of this increase reflects increased testing - the more you test, the more you diagnose.[13] Testing rates have increased from 1% in 2000 to 3.5 % in 2010[14] and current trends suggest over 100,000 cases will be diagnosed annually within 5 years. Analyses of sentinel clinic data show that the prevalence of infection in the population is increasing at a rate of 12% per year.[15] Chlamydia can have considerable adverse health consequences particularly for women: up to two thirds of tubal factor infertility and one third of ectopic pregnancy may be due to past infection.[16] This highlights that the burden of chlamydia will continue to increase in Australia.

Repeat chlamydia infections are very common and drive chlamydia transmission

Repeat chlamydia infections are common following treatment and account for a substantial proportion of incident infections. Several studies have reported alarmingly high chlamydia repeat infection rates among young women tested again post treatment. An Australian cohort of 1116

[1] Worm et al. Genituirn Med 1995; 71:244-246

young women found that among those women testing positive at recruitment, 18% tested positive again at 3 months (95%CI:8%, 34%) following treatment.[17] In the UK, a prospective cohort of 16 to 24 year old women from general practice reported a repeat infection rate of 29.9% per year after treatment (95% CI: 19.7, 45.4).[18] Another cohort of adolescent women in the US reported a repeat infection rate of 34% per year.[9] Although re-testing at 3 months after a positive diagnosis is recommended in Australia, re-testing rates remain low. A recent analysis of surveillance data found that only 14% are retested within 4 months following a positive diagnosis.[19] This suggests that there are many more repeat infections that go undetected in the population, leading to further transmission. **If we genuinely want to better control this chlamydia, then we need to better understand whether these are true re-infections or treatment failures so that we can further enhance partner notification or change treatment regimens.**

Repeat infections may represent treatment failure

Repeat infections may represent: 1) re-infection due to unprotected sexual contact with an infected partner; 2) treatment failure as a result of noncompliance with treatment, poor absorption of the drug, reduced antimicrobial susceptibility or antimicrobial resistance; or 3) persistence due to host factors such as immune response or other undefined host factors.[2-5 20] While most repeat infections are generally considered to be re-infections through exposure to an infected partner, emerging evidence suggests that *treatment failure* may account for a significant proportion. A partner treatment study found that among female participants who reported no sexual intercourse after treatment, 22 of 289 (8%; 95%CI: 5%, 11%) had persistent infection at follow up, suggestive of treatment failure.[8] A cohort of adolescent females also found a treatment failure rate of 7.9% (95%CI: 4%, 10.1%).[9] There is also in vitro evidence that chlamydia can enter a persistent form where infected cells exposed to β-lactam antibiotics, interferon-γ or deprived of iron supplements or amino acids can exhibit persistence.[2 3 5 21 22] This allows chlamydia to remain dormant, non-infectious and undetectable by culture but, on removing the stressful conditions, it can be recovered by culture. There is also evidence that latent infection may not be detectable, even using PCR, if only cells shed from the mucosal surface are sampled.[6 22 23] It is not known how often this persistent state persists in vivo and whether removal of treatment can trigger reactivation.[2-4 20]

Distinguishing between treatment failure and re-infection is vital for chlamydia control

Distinguishing between treatment failure and re-infection is important to focus treatment recommendations and infection control mechanisms. For example, if many repeat infections are due to antibiotic treatment failure, then international recommendations on chlamydia treatment need to be re-evaluated. If most are re-infections, then strategies to expedite partner treatment are necessary. Treatment failure will lead to persistent infection and if left untreated, chlamydia infection can last for several years.[24] This increases a woman's risk of developing further chlamydia sequelae and contributes to continued transmission in the population.

There are concerns about the evidence supporting current chlamydia treatment regimens

The recommended first line treatment for uncomplicated genital chlamydia infection in Australia is a 1gram dose of the macrolide antibiotic, azithromycin.[25] While doxycline 100mg twice daily for 7 days is a second line treatment for uncomplicated chlamydia in Australia, it is not widely used because there are concerns about compliance given the longer duration of treatment.[26] A meta-analysis of chlamydia treatment reported a 97% cure rate for azithromycin and 98% for doxycycline.[10] However, of the 12 trials included in this meta-analysis, 11 used culture or immunoassay rather than the more sensitive PCR to determine microbial cure at study end and

one third had significant attrition rates of at least 25%.[10] Further, three of the studies included in the meta-analysis had cure rates of 81-92% for azithromycin compared with 99-100% for doxycline. Given the use of culture rather than PCR, it is likely that the treatment efficacies in the treatment trials were over-estimated.[6 7 23]

Research methodology

Sampling frame

Women aged 18 years recruited via word of mouth, advertising on the University of Melbourne Staff News email or as patients attending at Melbourne Sexual Health Centre (MSHC, Victoria)
We aim to recruit 12-20 women.

Inclusion criteria

- Women aged 18 years and older
- Must have adequate English and comprehension skills to give informed consent.
- Able to attend the clinic at regular intervals during the study to collect and drop of specimens.

Exclusion criteria

- Pregnant women
- Women currently menstruating
- Women on medication likely to significantly interact with Azithromycin eg. Cyclosporin, digoxin
- Women known to have any allergies associated with macrolides (very rare).

Recruitment

Interested women and eligible women attending MSHC as a patient will be seen by the research assistant. The study will be advertised in the Staff News email at the University of Melbourne and by clinical staff at MSHC.

The research assistant will explain the study to them, assess for eligibility and take informed consent. The research assistant will explain how to collect specimens for investigation and will take a blood specimen.

A small number of women who have tested positive for chlamydia will also be recruited when they return to the clinic for treatment.

All participants will be given a 1 gram stat dose of azithromycin to take at recruitment regardless of whether or not they test positive for chlamydia. Please note: azithromycin is a very well tolerated and relatively benign antibiotic. It is recommended by the World Health Organisation for the 'presumptive' treatment of STIs for over a decade now[2] (2) Azithromycin has also been available without a prescription in the UK since 2008[3] and (3) has negligible, clinically significant drug interactions[4] and minimal/mild side effects[5]. It is also regular given to patients who have contact with chlamydia regardless of whether or not they are diagnosed with the infection.

---

[2] World Health Organisation, 2008 - Periodic presumptive treatment for sexually transmitted infections. Experience from the field and recommendations for research. *Presumptive is defined as a one-time treatment for a presumed infection in a person, or a group of people, at high risk of infection*

[3] http://www.mhra.gov.uk/NewsCentre/Pressreleases/CON023105

[4] Rapp R. Pharmacokinetics and Pharmacodynamics of Intravenous and Oral Azithromycin: Enhanced Tissue Activity and Minimal Drug Interactions. *Ann Pharmacother* 1998;32:785-93.

Specimen collection

Women will be asked to use self collected swabs and/or tampons to collect high vaginal material. These specimens will be immediately placed a solution containing methanol and placed at -80C within 4 hours following collection. Women will be asked to return to the clinic at regular time intervals to self collect specimens.

Specimen testing

Swabs, tampons and blood will be processed at the laboratory. Azithromycin levels as well as its metabolites will be measured using LCMS (Liquid Chromatographic Mass Spectrometry) at Metabolomics, Bio21, University of Melbourne.

Follow up

Participants will be telephoned regularly over the 7 days to remind them to drop off specimens.

Women diagnosed with chlamydia at recruitment, will be re-tested again at 3 months and treated again if chlamydia positive again.

Women will be reimbursed with up to a $100 voucher to cover their transport and time costs during the study.

Data collection

Participants will be asked to provide the following data:

Age, height, weight, any medications, use of vaginal lubricant or other gels, contact details.

Analysis

Absorption concentration over time will be monitored for each participant. Correlations with weight and height and medications will be assessed.

[5] Zuckerman JM et al. Macrolides, Ketolides, and Glycylcyclines: Azithromycin, Clarithromycin, Telithromycin, Tigecycline. *Infect Dis Clin N Am* 23 (2009) 997–1026. *The most common adverse effects reported with azithromycin were diarrhea (3.6%), nausea (2.6%), abdominal pain (2.5%), and headache or dizziness (1.3%)*

References

1. Alexander S, Pitt R, Horner P, Ison C. Antimicrobial resistance in Chlamydia trachomatis: is it a reality? *HIV Med* 2010;11(Suppl 1):15-16.
2. Wyrick PB. Chlamydia trachomatis persistence in vitro: an overview. *JID* 2010;201(S2):S88-S95.
3. Beatty WL, Morrison RP, Byrne GI. Reactivation of persistent Chlamydia trachomatis infection in cell culture. *Infection and Immunity* 1995;63(1):199-205.
4. Wang SA, Papp JR, Stamm WE, Peeling RW, Martin DH, Holmes KK. Evaluation of antimicrobial resistance and treatment failures for Chlamydia trachomatis: A meeting report. *JID* 2005;191(6):917-23.
5. Sandoz KM, Rockey DD. Antibiotic resistance in Chlamydiae. *Future Microbiol.* 2010;5(9):1427-42.
6. Horner P. The case for further treatment studies of uncomplicated genital Chlamydia trachomatis infection. *Sex. Transm. Inf.* 2006;82:340-43.
7. Handsfield HH. Questioning azithromycin for chlamydial infection. *Hunter Handsfielf's Personal Blog*: STD Prevention Online, 2010.
8. Golden MR, Whittington WL, Handsfield HH, Hughes JP, Stamm WE, Hogben M, et al. Effect of expedited treatment of sex partners on recurrent or persistent gonorrhoea or chlamydial infection. *N Engl J Med* 2005;352:676-85.
9. Batteiger BE, Tu W, Ofner S, Van Der Pol B, Stothard DR, Orr DP, et al. Repeated Chlamydia trachomatis genital infections in adolescent women. *J Infect Dis* 2010;201(1):42-51.
10. Lau C-Y, Qureshi AK. Azithromycin Versus Doxycycline for Genital Chlamydial Infections. A Meta-Analysis of Randomised Clinical Trials. *Sex Transm Dis* 2002;29(9):497-502.
11. Number of notifications of Chlamydial infections, Australia, 2009 by age group and sex. In: System NNDS, editor: Department of Health and Ageing, 2010.
12. (CDC) CfDCaP. Chlamydia - Women - Reported Cases and Rates by State/Area and Region in Alphabetical Order, United States and Outlying Areas, 2005-2009, 2010.
13. Hocking J, Fairley CK, Counahan M, Crofts N. The pattern of notification and testing for genital *Chlamydia trachomatis* infection in Victoria, 1998-2000: an ecological analysis. *Aust N Z J Public Health* 2003;27(4):405-08.
14. Medicare Australia. Medical Benefits Schedule Item Statistics 69316, 69317, 69319 2010.
15. O'Rouke K, Fairley C, Samaranayake A, Collingnon P, Hocking J. Trends in chlamydia positivity over time among women in Melbourne Australia, 2003-2007. *Sex Transm Dis* 2009;36(12):763-67.
16. Peipert JF. Genital Chlamydial Infections. *N Engl J Med* 2003;349(25):2424-30.
17. Walker J, Fairley C, Urban E, Chen MY, Bradshaw C, Walker S, et al. Maximising retention in a longitudinal study of sexually transmitted infections among young women in Australia. *BMC Public Health* 2011;Accepted February 2011.
18. LaMontagne D, Baster K, Emmett L, Nichols T, Randall S, McLean L, et al. Incidence and reinfection rates of genital chlamydia infection among women aged 16 to 24 years attending general practice, family planning and genitourinary medicine clinics in England: a prospective cohort study by the Chlamydia Recall Study Advisory Group. *Sex. Transm. Inf.* 2007;83:282-303.
19. Guy R, Wand H, Franklin N, Fairley C, Chen M, O'Connor C, et al. Re-testing for chlamydia at sexual health services in Australia, 2004-2008. *Sex Health* 2010;Accepted September.
20. Suchland RJ, Geisler WM, Stamm WE. Methodologies and cell lines used for antimicrobial susceptibility testing of Chlamydia spp. *Antimicrob. Agents Chemother.* 2003;47(2):636-42.
21. Harper A, Pogson CI, Jones ML, Pearce JH. Chlamydial Development Is Adversely Affected by Minor Changes in Amino Acid Supply, Blood Plasma Amino Acid Levels, and Glucose Deprivation. *Infect. Immun.* 2000;68(3):1457-64.
22. Hogan RJ, Mathews SA, Mukhopadhyay S, Summersgill JT, Timms P. Chlamydial Persistence: beyond the biphasic paradigm. *Infection and Immunity* 2004;72(4):1843-55.
23. Dean D, Suchland RJ, Stamm WE. Evidence for long-term cervical persistence of Chlamydia trachomatis by omp1 genotyping. *J Infect Dis* 2000;182(3):909-16.
24. Fairley C, Walker J, Gurrin L, Hocking J. Doctor, how long has my chlamydia been there? Answer: years. *Sex Transm Dis* 2007;34 (9):727-28.
25. Victoria SHSo. *National Management Guidelines for Sexually Transmissible Infections*. Carlton: Sexual Health Society of Victoria, 2008.
26. Bachmann LH, Stephens J, Richey CM, Hook EW. Measured versus self-reported compliance with doxycycline therapy for chlamydia-associated syndromes - High therapeutic success rates despite poor compliance. *Sex Transm Dis* 1999;26(5):272-78.

Questionnaire:

1) What is your age?
2) What is your height?
3) What is your weight?
4) Please list any medications you are currently taking, including any contraceptive medication
5) Are you using any vaginal lubricants or other gels?

Advertisement for University of Melbourne Staff News:

Women are sought to participate in a research project being conducted at the Melbourne School of Population Health that aims to determine the optimal sampling and testing method to measure absorption of the antibiotic azithromycin in the female genital tract.

Azithromycin is the current treatment in Australia for genital chlamydia infection, Australia's most common sexually transmitted infection currently infecting about 4% of young men and women.

There is some concern that this treatment may not always work at curing chlamydia and we want to determine whether treatment failure may be due to poor absorption of the drug.

Women will be asked to take 1 gram of azithromycin and then collect up to 7 high vaginal swabs and/or tampon specimens over a 7 day period. They will also provide a single blood specimen.

Azithromycin is a widely used drug with minimal side effects.

Participants will be reimbursed up to $100 for their time and contribution to this study.

Please call XXXXX if you are interested in participating and want further information.
