# Supplementary material for: Measurement of tissue azithromycin levels in self-collected vaginal swabs post treatment using liquid chromatography and tandem mass spectrometry (LC-MS/MS)
Source: PLoS One. 2017 May 12;12(5):e0177615. doi: 10.1371/journal.pone.0177615 (PMC5428968; doi:10.1371/journal.pone.0177615)
Supplement: S4 File — Alfred Hospital Ethics Approval Certificate for Clinical Trial Protocol 1. (PDF) [file pone.0177615.s004.pdf]

TheAlfred

ETHICS COMMITTEE CERTIFICATE OF APPROVAL

This is to certify that

Project No: 480/11

Project Title: Development of a standard method for measurement of azithromycin concentration in cervical cellular material – a pilot study

Principal Researcher: A/Professor Jane Hocking

Protocol No: Project 480/11 **dated:** Not specified

Participant Information and Consent Form Version 5.0 **dated:** 02-Mar-2012
Participant Information and Consent Form – Chlamydia Positive Version 5.0 **dated:** 02-Mar-2012

*was considered by the Ethics Committee on **22-Dec-2011**, meets the requirements of the National Statement on Ethical Conduct in Human Research (2007) and was **APPROVED** on **10-Apr-2012***

It is the Principal Researcher's responsibility to ensure that all researchers associated with this project are aware of the conditions of approval and which documents have been approved.

The Principal Researcher is required to notify the Secretary of the Ethics Committee, via amendment or progress report, of

- Any significant change to the project and the reason for that change, including an indication of ethical implications (if any);
- Serious adverse effects on participants and the action taken to address those effects;
- Any other unforeseen events or unexpected developments that merit notification;
- The inability of the Principal Researcher to continue in that role, or any other change in research personnel involved in the project;
- Any expiry of the insurance coverage provided with respect to sponsored clinical trials and proof of re-insurance;
- A delay of more than 12 months in the commencement of the project; and,
- Termination or closure of the project.

Additionally, the Principal Researcher is required to submit

- A Progress Report on the anniversary of approval and on completion of the project *(forms to be provided)*;

The Ethics Committee may conduct an audit at any time.

All research subject to the Alfred Hospital Ethics Committee review must be conducted in accordance with the National Statement on Ethical Conduct in Human Research (2007).

The Alfred Hospital Ethics Committee is a properly constituted Human Research Ethics Committee in accordance with the National Statement on Ethical Conduct in Human Research (2007).

SPECIAL CONDITIONS

None

SIGNED:

R Frew
Secretary, Ethics Committee

Please quote project number and title in all correspondence
