# Supplementary material for: Measurement of tissue azithromycin levels in self-collected vaginal swabs post treatment using liquid chromatography and tandem mass spectrometry (LC-MS/MS)
Source: PLoS One. 2017 May 12;12(5):e0177615. doi: 10.1371/journal.pone.0177615 (PMC5428968; doi:10.1371/journal.pone.0177615)
Supplement: S7 File — Second amended clinical trial protocol submitted to and approved by the Alfred Hospital Ethics Committee (labelled version 5). (PDF) [file pone.0177615.s007.pdf]

Protocol

Aim: To measure azithromycin concentrations over the course of 24 hours in vaginal-cervicalcellular material in women following a single 1 gram dose.

Rationale:

- There is considerable concern internationally about chlamydia treatment failure.[1-7]
- Studies have found that chlamydia treatment failure may be at least 8%,[8 9] considerably higher than the 2-3%[10] failure expected based on results of previous chlamydia treatment trials that with the exception of one trial, all used the less sensitive culture method to measure antimicrobial cure.
- A treatment failure rate of 8% rather than 2-3% means a further nearly 3,000[11] in Australia and 70,000[12] women in the USA were inadequately treated for chlamydia in 2009, leading to longer duration of infection, increased risk of developing sequelae and continued transmission.
- **It remains uncertain if this treatment failure is because of (1) re-infection by an untreated partner; (2) failure of the drug itself due to poor absorption from the stomach or low concentrations in female genital tissue where it is needed to work, or; (3) the body's immune system's inability to fight the infection.**
- We have received NHMRC funding to conduct a large cohort study of 450 women that aims to estimate azithromycin treatment failure for genital chlamydia infection. In order to understand failure due to reason (2) above (poor absorption), we propose to develop a test to measure the concentration of the drug in female cervical cellular material. We wish to determine whether azithromycin is present at the cervix in sufficient concentrations to treat chlamydia. This was done by Worm and colleagues in the early 1990s1 on mucus aspirated from the cervix, a method unlikely to be acceptable to women today as part of a large cohort study.
- **We have previously developed an assay to detect azithromycin in self collected vaginal swabs (ETHICS NUMBER: 480/11) and seek now to extend that study to measure azithromycin concentrations in women up to 24 hours after treatment.**
- **This proposal is an expansion on our previously approved project. We propose to collect a rapid series of vaginal swabs from 10-20 women aged at least 18 years of age over the course of 24 hours and measure azithromycin concentrations using our validated test.**
- We would also like to conduct a sub-study comparing collection sites for chlamydia culture. We would like to compare high vaginal samples with endocervical samples from the same woman. If we are able to show that a high vaginal sample is sufficient for chlamydia culture then we can avoid the need for a speculum examination for women participating in the cohort study. **This will be a sub-study and not a requirement for participation in the azithromycin concentration study.**

Background:

Chlamydia rates continue to increase

Worldwide, chlamydia diagnosis rates have increased dramatically over the last decade – it is the most commonly diagnosed bacterial STI throughout the developed world. Chlamydia diagnosis rates have increased by over 400% in the last decade in Australia with 72,123 cases diagnosed in 2010.[11] Part of this increase reflects increased testing - the more you test, the more you diagnose.[13] Testing rates have increased from 1% in 2000 to 3.5 % in 2010[14] and current trends suggest over 100,000 cases will be diagnosed annually within 5 years. Analyses of sentinel clinic

[1] Worm et al. Genituirn Med 1995; 71:244-246

data show that the prevalence of infection in the population is increasing at a rate of 12% per year.[15] Chlamydia can have considerable adverse health consequences particularly for women: up to two thirds of tubal factor infertility and one third of ectopic pregnancy may be due to past infection.[16] This highlights that the burden of chlamydia will continue to increase in Australia.

Repeat chlamydia infections are very common and drive chlamydia transmission
Repeat chlamydia infections are common following treatment and account for a substantial proportion of incident infections. Several studies have reported alarmingly high chlamydia repeat infection rates among young women tested again post treatment. An Australian cohort of 1116 young women found that among those women testing positive at recruitment, 18% tested positive again at 3 months (95%CI:8%, 34%) following treatment.[17] In the UK, a prospective cohort of 16 to 24 year old women from general practice reported a repeat infection rate of 29.9% per year after treatment (95% CI: 19.7, 45.4).[18] Another cohort of adolescent women in the US reported a repeat infection rate of 34% per year.[9] Although re-testing at 3 months after a positive diagnosis is recommended in Australia, re-testing rates remain low. A recent analysis of surveillance data found that only 14% are retested within 4 months following a positive diagnosis.[19] This suggests that there are many more repeat infections that go undetected in the population, leading to further transmission. **If we genuinely want to better control this chlamydia, then we need to better understand whether these are true re-infections or treatment failures so that we can further enhance partner notification or change treatment regimens.**

Repeat infections may represent treatment failure
Repeat infections may represent: 1) re-infection due to unprotected sexual contact with an infected partner; 2) treatment failure as a result of noncompliance with treatment, poor absorption of the drug, reduced antimicrobial susceptibility or antimicrobial resistance; or 3) persistence due to host factors such as immune response or other undefined host factors.[2-5 20] While most repeat infections are generally considered to be re-infections through exposure to an infected partner, emerging evidence suggests that *treatment failure* may account for a significant proportion. A partner treatment study found that among female participants who reported no sexual intercourse after treatment, 22 of 289 (8%; 95%CI: 5%, 11%) had persistent infection at follow up, suggestive of treatment failure.[8] A cohort of adolescent females also found a treatment failure rate of 7.9% (95%CI: 4%, 10.1%).[9] There is also in vitro evidence that chlamydia can enter a persistent form where infected cells exposed to β-lactam antibiotics, interferon-γ or deprived of iron supplements or amino acids can exhibit persistence.[2 3 5 21 22] This allows chlamydia to remain dormant, non-infectious and undetectable by culture but, on removing the stressful conditions, it can be recovered by culture. There is also evidence that latent infection may not be detectable, even using PCR, if only cells shed from the mucosal surface are sampled.[6 22 23] It is not known how often this persistent state persists in vivo and whether removal of treatment can trigger reactivation.[2-4 20]

Distinguishing between treatment failure and re-infection is vital for chlamydia control
Distinguishing between treatment failure and re-infection is important to focus treatment recommendations and infection control mechanisms. For example, if many repeat infections are due to antibiotic treatment failure, then international recommendations on chlamydia treatment need to be re-evaluated. If most are re-infections, then strategies to expedite partner treatment are necessary. Treatment failure will lead to persistent infection and if left untreated, chlamydia infection can last for several years.[24] This increases a woman's risk of developing further chlamydia sequelae and contributes to continued transmission in the population.

There are concerns about the evidence supporting current chlamydia treatment regimens
The recommended first line treatment for uncomplicated genital chlamydia infection in Australia is a 1gram dose of the macrolide antibiotic, azithromycin.[25] While doxycline 100mg twice daily for 7 days is a second line treatment for uncomplicated chlamydia in Australia, it is not widely used because there are concerns about compliance given the longer duration of treatment.[26] A meta-analysis of chlamydia treatment reported a 97% cure rate for azithromycin and 98% for doxycycline.[10] However, of the 12 trials included in this meta-analysis, 11 used culture or immunoassay rather than the more sensitive PCR to determine microbial cure at study end and one third had significant attrition rates of at least 25%.[10] Further, three of the studies included in the meta-analysis had cure rates of 81-92% for azithromycin compared with 99-100% for doxycline. Given the use of culture rather than PCR, it is likely that the treatment efficacies in the treatment trials were over-estimated.[6 7 23]

Research methodology
Sampling frame
Female patients attending the Melbourne Sexual Health Centre (MSHC) and receiving azithromycin 1g as treatment for confirmed or suspected chlamydia will be recruited. Recruitment will be conducted by trained research staff (Karen Worthington). Women will be informed of the possibility of a study when they are given their result over the phone, or when a clinician has decided that treatment for chlamydia is indicated. They will be given an appointment to see one of the trained research staff to receive treatment. At their appointment they will be informed of the study and given the option to participate. If they do not wish to participate they will receive standard care as delivered by the clinic. Refusing to participate will not affect their care.

Female volunteers, not attending MSHC for chlamydia treatment or suspected chlamydia, aged 18 years or older will be recruited via word of mouth, advertising on the University of Melbourne staff newsletter or MSHC staff newsletter.

Once consented, patients will be asked to take a 1g dose of azithromycin and provide up to 11 specimens, over the following 5 hours, and a final specimen 24 hours after taking the azithromycin. At the baseline assessment the research nurse will collect one high vaginal swab for culture, and then perform a speculum examination to collect an endocervical swab for culture. This will be optional, and those women who do not wish to have a speculum examination can still participate in the study. The remaining self-collected high vaginal specimens will be collected at baseline (before treatment) and then every 30 mins for the next 5 hours. Patients will be asked to return to the clinic the next day to provide a final self-collected specimen. The specimens will be collected and placed in cold methanol solution immediately by the research nurse. In addition, a 4 ml blood specimen will be collected at the 4 hour mark using standard venepuncture to ascertain whether the azithromycin has been absorbed through the stomach.

We aim to recruit 10-20 women.

Inclusion criteria
- Women aged 18 years and older

- Either attending MSHC and receiving 1g azithromycin as treatment for confirmed chlamydia, or receiving 1g azithromycin for treatment of suspected chlamydia or happy to receive 1g azithromycin as part of the study.
- Must have adequate English and comprehension skills to give informed consent.
- Able to attend the clinic at regular intervals during the study to collect and drop of specimens.

Exclusion criteria

- Pregnant women
- Women currently menstruating
- Women on medication likely to significantly interact with Azithromycin eg. Cyclosporin, digoxin
- Women known to have any allergies associated with macrolides (very rare).

Recruitment

Female patients who have attended MSHC and have a positive chlamydia result are routinely called to come back for treatment. Eligible women will be told over the phone that we are currently running a study about azithromycin treatment and that if they would like to participate, they will be asked to stay remain in the vicinity of the clinic for 5 hours. They will be told that they do not have to make a decision yet, and that if they do not wish to consent, their care will not be altered. Female patients who attend MSHC who have been prescribed azithromycin 1g for suspected chlamydia will also be given the option to participate. Interested women will be seen by the research assistant.

In addition, interested female volunteers will be able to contact the research nurse and enquire about participation if they hear about the study either by word of mouth, or in MSHC or University Staff News email.

The research assistant will explain the study to all potential participants, assess for eligibility and take informed consent. If the patient does not wish to participate, the research nurse will treat them as is routine practice. If they consent, the research nurse will collect one high vaginal swab for culture, and then perform a speculum examination to collect an endocervical swab for culture. These two samples will be optional, and those women who do not wish to have a speculum examination can still participate in the study. If they decline the speculum or are volunteers, participants will self-collect all high vaginal samples.The research nurse will explain how to collect specimens for investigation. They will be given a baseline swab to collect and a 1g dose of azithromycin. The research nurse will then arrange for them to return to the clinic every 30 mins to take a repeat specimen and will also take the blood specimen 4 hours after their dose of azithromycin.

All participants will be given a 1 gram stat dose of azithromycin to take at recruitment regardless of whether or not they test positive for chlamydia. Please note: azithromycin is a very well tolerated and relatively benign antibiotic. It is recommended by the World Health Organisation for the 'presumptive' treatment of STIs for over a decade now[2] (2) Azithromycin has also been available without a prescription in the UK since 2008 and is also given to sexual partners

[2] World Health Organisation, 2008 - Periodic presumptive treatment for sexually transmitted infections. Experience from the field and recommendations for research. *Presumptive is defined as a one-time treatment for a presumed infection in a person, or a group of people, at high risk of infection*

regardless of whether or not they have chlamydia diagnosed[3] and (3) has negligible, clinically significant drug interactions[4] and minimal/mild side effects[5]. It is also regularly given to patients who have contact with chlamydia regardless of whether or not they are diagnosed with the infection. Additionally the USA FDA has recently approved the labelling of Zithromax with the indication that "***Therapy with ZITHROMAX may be initiated before results of these tests are known*".**[6]

Azithromycin will be prescribed by a MSHC clinician and administered in consultation with the clinician in accordance with routine clinical care at MSHC. All participants will be asked to monitor adverse events and report them to the research assistant. They will receive a study phone number that they can call should they experience any adverse events. The research assistant will report to a safety and monitoring committee (including a senior sexual health physician from MSHC, the head of research/evaluation at MSHC and the chief investigator of the study). Any adverse events will be managed according to MSHC protocol.

Specimen collection
The research nurse will collect one high vaginal swab and one endocervical swab. Both of these swabs will go into transport medium for culture. They will be placed in -80C following collection. Women will be asked to use self-collected swabs to collect high vaginal material. These specimens will be immediately placed in cold 100% methanol and placed at -80C following collection. Women will be asked to return to the clinic every 30 mins to self-collect specimens for up to 10 samples over 5 hours, and then return to the clinic the next day to provide one more self-collected specimen. A blood sample will be collected by the research nurse 4 hours after taking azithromycin

Specimen testing
Swabs and blood will be processed at the laboratory. Azithromycin levels as well as its metabolites will be measured using LCMS (Liquid Chromatographic Mass Spectrometry) at Metabolomics, Bio21, University of Melbourne. Samples for culture will be sent to Queensland University of Technology for chlamydia culture.

Follow up
As participants will be followed up over the course of 24 hours, there will not be any additional follow up unless they would like to be informed of results.

Women diagnosed with chlamydia at recruitment, will be re-tested again at 3 months and treated again if chlamydia positive again as is routine practice at both clinics. Women attending as contacts of chlamydia, or receiving presumptive treatment for chlamydia will be informed of the result of their chlamydia test within 7 days.

---

[3] http://www.mhra.gov.uk/NewsCentre/Pressreleases/CON023105

[4] Rapp R. Pharmacokinetics and Pharmacodynamics of Intravenous and Oral Azithromycin: Enhanced Tissue Activity and Minimal Drug Interactions. *Ann Pharmacother* 1998;32:785-93.

[5] Zuckerman JM et al. Macrolides, Ketolides, and Glycylcyclines: Azithromycin, Clarithromycin, Telithromycin, Tigecycline. *Infect Dis Clin N Am* 23 (2009) 997–1026. *The most common adverse effects reported with azithromycin were diarrhea (3.6%), nausea (2.6%), abdominal pain (2.5%), and headache or dizziness (1.3%)*

[6] http://www.accessdata.fda.gov/drugsatfda_docs/label/2011/050693s017,050710s033,050711s031,050784s018,050730s026lbl.pdf (page 10) **LABEL APPROVED 28/1/2011**

Women will be reimbursed with up to a $75 voucher ($50 for the first 11 swabs, and $25 for the final swab 24 hours after the first) to cover their transport and time costs during the study. This will be provided to the women at the end of the study.

Data collection

Participants will be asked to provide the following data:

Age, height, weight, any medications, use of vaginal lubricant or other gels, contact details.

Analysis

Absorption concentration over time will be monitored for each participant. Correlations with weight and height and medications will be assessed.

References

1. Alexander S, Pitt R, Horner P, Ison C. Antimicrobial resistance in Chlamydia trachomatis: is it a reality? *HIV Med* 2010;11(Suppl 1):15-16.
2. Wyrick PB. Chlamydia trachomatis persistence in vitro: an overview. *JID* 2010;201(S2):S88-S95.
3. Beatty WL, Morrison RP, Byrne GI. Reactivation of persistent Chlamydia trachomatis infection in cell culture. *Infection and Immunity* 1995;63(1):199-205.
4. Wang SA, Papp JR, Stamm WE, Peeling RW, Martin DH, Holmes KK. Evaluation of antimicrobial resistance and treatment failures for Chlamydia trachomatis: A meeting report. *JID* 2005;191(6):917-23.
5. Sandoz KM, Rockey DD. Antibiotic resistance in Chlamydiae. *Future Microbiol.* 2010;5(9):1427-42.
6. Horner P. The case for further treatment studies of uncomplicated genital Chlamydia trachomatis infection. *Sex. Transm. Inf.* 2006;82:340-43.
7. Handsfield HH. Questioning azithromycin for chlamydial infection. *Hunter Handsfielf's Personal Blog*: STD Prevention Online, 2010.
8. Golden MR, Whittington WL, Handsfield HH, Hughes JP, Stamm WE, Hogben M, et al. Effect of expedited treatment of sex partners on recurrent or persistent gonorrhoea or chlamydial infection. *N Engl J Med* 2005;352:676-85.
9. Batteiger BE, Tu W, Ofner S, Van Der Pol B, Stothard DR, Orr DP, et al. Repeated Chlamydia trachomatis genital infections in adolescent women. *J Infect Dis* 2010;201(1):42-51.
10. Lau C-Y, Qureshi AK. Azithromycin Versus Doxycycline for Genital Chlamydial Infections. A Meta-Analysis of Randomised Clinical Trials. *Sex Transm Dis* 2002;29(9):497-502.
11. Number of notifications of Chlamydial infections, Australia, 2009 by age group and sex. In: System NNDS, editor: Department of Health and Ageing, 2010.
12. (CDC) CfDCaP. Chlamydia - Women - Reported Cases and Rates by State/Area and Region in Alphabetical Order, United States and Outlying Areas, 2005-2009, 2010.
13. Hocking J, Fairley CK, Counahan M, Crofts N. The pattern of notification and testing for genital *Chlamydia trachomatis* infection in Victoria, 1998-2000: an ecological analysis. *Aust N Z J Public Health* 2003;27(4):405-08.
14. Medicare Australia. Medical Benefits Schedule Item Statistics 69316, 69317, 69319 2010.
15. O'Rouke K, Fairley C, Samaranayake A, Collingnon P, Hocking J. Trends in chlamydia positivity over time among women in Melbourne Australia, 2003-2007. *Sex Transm Dis* 2009;36(12):763-67.
16. Peipert JF. Genital Chlamydial Infections. *N Engl J Med* 2003;349(25):2424-30.
17. Walker J, Fairley C, Urban E, Chen MY, Bradshaw C, Walker S, et al. Maximising retention in a longitudinal study of sexually transmitted infections among young women in Australia. *BMC Public Health* 2011;Accepted February 2011.
18. LaMontagne D, Baster K, Emmett L, Nichols T, Randall S, McLean L, et al. Incidence and reinfection rates of genital chlamydia infection among women aged 16 to 24 years attending general practice, family planning and genitourinary medicine clinics in England: a prospective cohort study by the Chlamydia Recall Study Advisory Group. *Sex. Transm. Inf.* 2007;83:282-303.
19. Guy R, Wand H, Franklin N, Fairley C, Chen M, O'Connor C, et al. Re-testing for chlamydia at sexual health services in Australia, 2004-2008. *Sex Health* 2010;Accepted September.
20. Suchland RJ, Geisler WM, Stamm WE. Methodologies and cell lines used for antimicrobial susceptibility testing of Chlamydia spp. *Antimicrob. Agents Chemother.* 2003;47(2):636-42.

21. Harper A, Pogson CI, Jones ML, Pearce JH. Chlamydial Development Is Adversely Affected by Minor Changes in Amino Acid Supply, Blood Plasma Amino Acid Levels, and Glucose Deprivation. *Infect. Immun.* 2000;68(3):1457-64.
22. Hogan RJ, Mathews SA, Mukhopadhyay S, Summersgill JT, Timms P. Chlamydial Persistence: beyond the biphasic paradigm. *Infection and Immunity* 2004;72(4):1843-55.
23. Dean D, Suchland RJ, Stamm WE. Evidence for long-term cervical persistence of Chlamydia trachomatis by omp1 genotyping. *J Infect Dis* 2000;182(3):909-16.
24. Fairley C, Walker J, Gurrin L, Hocking J. Doctor, how long has my chlamydia been there? Answer: years. *Sex Transm Dis* 2007;34 (9):727-28.
25. Victoria SHSo. *National Management Guidelines for Sexually Transmissible Infections*. Carlton: Sexual Health Society of Victoria, 2008.
26. Bachmann LH, Stephens J, Richey CM, Hook EW. Measured versus self-reported compliance with doxycycline therapy for chlamydia-associated syndromes - High therapeutic success rates despite poor compliance. *Sex Transm Dis* 1999;26(5):272-78.

Questionnaire:

1) What is your age?
2) What is your height?
3) What is your weight?
4) Please list any medications you are currently taking, including any contraceptive medication
5) Are you using any vaginal lubricants or other gels?
