# Supplementary material for: Measurement of tissue azithromycin levels in self-collected vaginal swabs post treatment using liquid chromatography and tandem mass spectrometry (LC-MS/MS)
Source: PLoS One. 2017 May 12;12(5):e0177615. doi: 10.1371/journal.pone.0177615 (PMC5428968; doi:10.1371/journal.pone.0177615)
Supplement: S8 File — Alfred Hospital Ethics Approval Certificate for amended protocol 3 (labelled version 5). (PDF) [file pone.0177615.s008.pdf]

TheAlfred

Ethics Committee

Certificate of Approval of Amendments

This is to certify that amendments to

Project: **480/11 Development of a standard method for measurement of azithromycin concentration in cervical cellular material – a pilot study**

Principal Researcher: **A/Professor Jane Hocking**

Protocol: **Project 480/11**
Amendment: **Protocol Version 5.0**
Protocol Amendment dated: **24-Jan-2014**
Participant Information & Consent Form: **Version 9** dated: **24-Jan-2014**

have been approved in accordance with your amendment application dated **28-Jan-2014** on the understanding that you observe the National Statement on Ethical Conduct in Human Research.

It is now your responsibility to ensure that all people associated with this particular research project are made aware of what has actually been approved and any caveats specified in correspondence with the Ethics Committee. Any further change to the application which is likely to have a significant impact on the ethical considerations of this project will require approval from the Ethics Committee.

Chair, Ethics Committee (or delegate)

Date: **07-Mar-2014**

R Frew
Secretary, Ethics Committee

All research subject to Alfred Hospital Ethics Committee review must be conducted in accordance with the National Statement on Ethical Conduct in Human Research (2007).

The Alfred Ethics Committee is a properly constituted Human Research Ethics Committee operating in accordance with the National Statement on Ethical Conduct in Human Research (2007).
